# Prediction of 10‐Year Fragility Fractures Using Muscle Health Indicators in Postmenopausal Women: The OsteoLaus Cohort

**DOI:** 10.1002/jcsm.13837

**Published:** 2025-06-04

**Authors:** Colin Vendrami, Elena Gonzalez Rodriguez, Guillaume Gatineau, Peter Vollenweider, Pedro Marques‐Vidal, Olivier Lamy, Didier Hans, Enisa Shevroja

**Affiliations:** ^1^ Interdisciplinary Center of Bone Diseases, Rheumatology Unit, Bone and Joint Department Lausanne University Hospital and University of Lausanne Lausanne Switzerland; ^2^ Department of Medicine, Internal Medicine Lausanne University Hospital and University of Lausanne Lausanne Switzerland

**Keywords:** dual‐x‐ray absorptiometry, epidemiology, fragility fracture, muscle, osteoporosis, population studies, sarcopenia

## Abstract

**Background:**

Muscle strength, mass and function have been associated with falls, fractures and mortality, but the results vary between previous studies. We aimed to investigate the predictive ability of muscle strength and mass with 10‐year incident fragility fractures.

**Methods:**

This study included 1475 postmenopausal women aged 50–80 years (OsteoLaus cohort, Lausanne, Switzerland). Handgrip strength (HGS) was assessed. With a Jamar dynamometer and lean mass (LM) with dual x‐ray absorptiometers (DXA) every 2.5 years for 10 years. LM, appendicular lean mass (ALM) and their indexes were assessed following the International Society for Clinical Densitometry (ISCD) guidelines. Main outcomes included hip, humerus and forearm low‐trauma fractures from in‐person interviews and vertebral fracture (VF) from lateral DXA screening. Secondary outcomes included falls and death. Baseline values were compared using two‐sided *t*‐test or Wilcoxon test (*p* < 0.0029 based on Bonferroni). Multivariate analysis included time to fracture with accelerated failure time (AFT) model and odds ratio (OR) with logistic regression, 95% confidence interval (CI) and C‐Index or AUC.

**Results:**

After 10.2 ± 0.4 years of follow‐up, 944 women remained enrolled (age 73.0 ± 6.9 years, BMI 25.7 ± 4.8 kg/m^2^, ALM 16.8 ± 2.5 kg, HGS 21.2 ± 5.5 kg), of whom 260 fractured (174 VF, 107 non‐VF), 863 fell and 74 died. Participants with an incident fragility fracture had a 1.5‐kg lower HGS at baseline but no significant difference in their ALM, ALM/height^2^ and ALM/BMI compared to nonfractured participants. In the multivariable models, one SD increase in ALM (+2.58 kg) was associated with a 0.72 (CI:0.61–0.85) and 0.67 (CI:0.55–0.82) shorter time to major osteoporotic fractures (MOF) and VF. While ALM/BMI was associated with a 1.26 (CI:1.01–1.59) and 1.98 (CI:1.21–3.25) longer time to MOF and non‐VF. One SD increase in HGS was associated with a 1.37 (CI:1.03–1.81) longer time to non‐VF only. A careful consideration of body weight and fat mass is needed in the association of lean mass with fractures. Baseline muscle parameters were not different for participant with or without incident fall or death.

**Conclusions:**

Lean mass and grip strength appear as independent risk factors for incident MOF, but with limited additional prediction performance. The prediction of fragility fractures differs between the fracture sites. Further studies with larger sample size, other muscle assessment modalities considering weight or fat mass as covariate, and broader ethnicities are needed.

## 1 Introduction

The 21st century is marked by a rapid worldwide population ageing, increasing the burden of chronic diseases such as osteoporosis—bone fragility [1]. In Europe, the costs of fragility fractures raised from EUR 37.4 in 2010 to EUR 56.9 billion in 2019 [2]. Clinically, a fracture is considered as osteoporotic when secondary to low‐trauma, such as the shock after a fall from a standing height or less [3]. During a lifetime, one‐third of women and one‐sixth of men will sustain a fragility fracture, which induces pain, disability, mobility reduction and increased mortality [2]. Multiple bone remodelling targeted drugs are efficient in improving bone resistance and reducing the risk of fragility fractures [4]. The main challenge lies in the accurate prediction of fracture risk and the eventual treatment of individuals at high risk for fracture [2, 5] as over half of the patients sustaining fractures do not have densitometric osteoporosis [6]. Fracture risk prediction scores have been developed by considering the multifactorial and age‐related pathophysiology of fragility fractures, the most widely used tool being FRAX [6].

Alongside age‐related bone deterioration, muscle decreases by 40% of its volume between the age of 20 and 80 years [7]. In the late 1980s, the loss of muscle mass with age was first described as sarcopenia by Rosenberg [8]. The sarcopenia definition now includes a composite deterioration of various muscle health indicators, such as muscle mass, strength and function, whose decline is associated with falls, fractures, hospitalization and mortality [8, 9]. In a 2024 scoping review including 67 studies and 2.8 million person‐years, the measures of muscle function and strength, but not of muscle mass, were predictive of incident fragility fracture [10]. As the results vary between studies, further evidence is needed to map the pathophysiological link between muscle mass and the adverse events of sarcopenia [10].

The main objective of this study was to analyse the ability of muscle strength and mass to predict the 10‐year incidence of fragility fractures and to investigate their eventual independent and incremental value in a prediction model with known risk factors. The secondary objectives were to analyse these associations for shorter follow‐up periods of 2.5 and 5 years and to assess the association of muscle parameters with falls, slow gait speed and death.

## 2 Material and Methods

The OsteoLaus study received approval from the Institutional Ethics Committee of the University of Lausanne, and all participants signed informed consent (reference 215/09). This article follows the STROBE statement (see Checklist S1).

### 2.1 Cohort and Study Design

This analysis was embedded in the OsteoLaus Study, a substudy of the CoLaus|PsyCoLaus study. Data collection was performed within the CoLaus|PsyCoLaus and OsteoLaus visits (https://www.colaus‐psycolaus.ch/). CoLaus is an ongoing prospective cohort initiated in 2003, studying the determinants of cardiovascular and psychiatric diseases in 6733 men and women aged 35 to 75 years from Lausanne, Switzerland [11]. The OsteoLaus study follows the same prospective design and included CoLaus women aged between 50 and 80 years to study their bone health [12]. The OsteoLaus first visit (baseline, 2010–2012) included 1475 women, of whom 98.4% were Caucasian. Follow‐up visits took place each 2.5 years: second (2012–2015 *n* = 1349), third (2015–2018, *n* = 1242), fourth (2017–2020, *n* = 1104) and fifth (2020–2022, *n* = 944) visits. At each OsteoLaus visit, the number of participants decreased due to loss of follow‐up, death, institutionalization, refusal, body mass index (BMI) over 40 kg/m^2^ or severe psychiatric disease.

### 2.2 Primary Outcome: Fragility Fractures

The main outcomes were incident major osteoporotic fractures (MOF), vertebral fractures (VF) and non‐VF (hip, humerus and forearm). An incident MOF was defined as a low‐trauma VF (Grades II and III), hip, forearm and proximal humerus fracture that occurred within the follow‐up. Two Grade I VF were also considered as one Grade II. Data on the MOF site, date and mechanism were collected during in‐person questionnaires at each OsteoLaus visit. The occurrence of new radiological VF was assessed by the dual x‐ray absorptiometers (DXA)‐derived lateral image of the spine (VF assessment) at each visit. Each VF was graded with the semiquantitative Genant method [13]. All VF were assessed independently by two different experienced physicians and adjudicated by a third one when the results differed.

### 2.3 Secondary Outcomes: Falls, Gait Speed and Mortality

Data on falls were collected with in‐person questionnaires (yes/no), with approximately 30% of women reporting a fall since the previous visit at each study visit (Table 1).

**TABLE 1:** Participants' characteristics through each OsteoLaus follow‐up.

	Baseline	2.5 years	5 years	7.5 years	10 years
Sample size	1475	1349	1242	1104	947
Visit dates (first–last)	03.2010–12.2012	09.2012–06.2015	04.2015–01.2018	11.2017–10.2020	03.2020–10.2022
Age (years)	64.5 ± 7.6	67.1 ± 7.5	69.2 ± 7.4	71.4 ± 7.2	73.0 ± 6.9
BMI (kg/cm^2^)	25.9 ± 4.5	26.0 ± 4.6	26.1 ± 4.7	25.9 ± 4.8	25.7 ± 4.8
Appendicular lean mass (kg.)	17.1 ± 2.6^H^	17.4 ± 2.7^H^	16.9 ± 2.6^L^	16.8 ± 2.6^L^	16.8 ± 2.5^L^
Handgrip strength (kg.)	23.9 ± 5.9	N.A.	24.1 ± 5.7	23.0 ± 6.0	21.2 ± 5.5
Femoral neck BMD T‐score	−1.1 ± 1.0	−1.1 ± 1.0	−1.2 ± 0.9	−1.3 ± 0.9	−1.4 ± 0.9
Recent falls (yes/no)	0.3 ± 0.4	0.3 ± 0.4	0.3 ± 0.5	0.3 ± 0.5	0.3 ± 0.5
6‐m gait speed (m/s)	N.A.	N.A.	N.A.	1.1 ± 0.2	1.1 ± 0.2
Follow‐up duration (years)	N.A.	2.6 ± 0.3	5.1 ± 0.3	7.6 ± 0.3	10.2 ± 0.4

*Note:* Body composition was measured with Hologic (^H^) the first two visits and lunar (^L^) the three last visits.

Abbreviations: BMD, bone mineral density; BMI, body mass index; N.A., not available.

Gait speed (m/s) was measured for a 6‐m walk distance at the patient's usual pace with its own auxiliary means (when needed) at fourth and fifth OsteoLaus visits only. Detailed and complete muscle assessments procedures were described previously [14]. All participants with normal gait speed (≥ 0.8 m/s) at Visit 4 and with a slow gait speed (< 0.8 m/s) at Visit 5 were considered as incident cases of low muscle function.

Death data were retrieved for each loss of follow‐up through patient medical records, publicly available death reports or were censored otherwise.

### 2.4 Handgrip Strength (HGS) and Lean Mass

HGS was measured four times: twice in the CoLaus Study visits, coinciding with the OsteoLaus baseline and third visits; and twice in the OsteoLaus fourth and fifth visits, using a JAMAR Baseline hydraulic hand dynamometer (Fabrication Enterprises, Inc., White Plains, NY, USA), following the American Society of Hand Therapists guidelines. The complete procedure has been described previously [14].

Details of body composition assessment by DXA in the OsteoLaus cohort have been described previously [15] and followed the International Society for Clinical Densitometry (ISCD) guidelines [16]. A Hologic QDR Discovery A device was used for the baseline and second visit, and a GE Lunar iDXA device for the three last visits. Lean mass measures were retrieved for each body region including total body, arms and legs. Appendicular lean mass (ALM) was calculated as the sum of both arms and legs. Lean percent was calculated as the regional lean mass divided by the regional total mass. Handgrip muscle‐specific strength was calculated by dividing HGS by the arm lean mass from the same side as the HGS test [8].

### 2.5 Covariables

All covariates were selected based on their association with fragility fractures, extracted from previous literature and from well‐studied risk factors of fragility fractures [6, 10]. Directed acyclic graphs (DAGs) were created to visually demonstrate this hypothetical relation from muscle parameters to fractures [17]. Age, height, weight and other risk factors (cf. below) were considered as confounding factors. Falls, bone mineral density (BMD) and muscle function (gait speed) were considered as mediators.

Height was measured using a portable stadiometer (Seca version 216, Seca, Chino, CA, USA) with 0.1‐cm precision. Weight was measured with participants barefoot and in medical coat, using an electronic scale with 0.1‐kg precision (Seca Clara 803, Seca, Chino, CA, USA). BMI represent the weight divided by height squared (kg/m^2^). Other risk factors, namely, smoking status, alcohol consumption and diabetes were derived from questionnaires data (yes/no). Femoral neck BMD and its T‐score were retrieved from DXA assessment at the left hip, following the ISCD guidelines [16]. The 10‐year risk of hip fracture or MOF was calculated with the FRAX calculator for cohort settings (frax.shef.ac.uk).

### 2.6 Statistical Analysis

The datasets and statistical analysis from the current study are not publicly available but can be shared upon reasonable request (https://www.colaus‐psycolaus.ch). Statistical analysis and data visualizations were performed with Python (v3.10.13) using the pandas (v2.1.4), seaborn (v0.12.2), statsmodels (v0.14.0), scypi.stats (v1.11.4) and lifelines (v0.28.0) libraries. The distribution and potential outliers of all included numerical variables were visually checked with boxplots, quantile‐quantile plots and assessed for normality using the Shapiro–Wilk test. For both the primary and secondary outcomes, we used *t*‐test or Wilcoxon–Mann–Whitney test for the comparison of means, based on the normal and nonnormal distribution, respectively. Chi‐squared test was used for dichotomous variables. The p‐value was considered as significant for a two‐sided test with 95% confidence interval (CI). The significance level was set to *p* < 0.0029 using the Bonferroni correction for multiple testing (considering the 17 variables tested upon one hypothesis as in Table 2, p‐value: 0.05/17 = 0.0029).

**TABLE 2:** Comparison of baseline covariates and muscle assessments between participants with or without fragility fractures, fall or death in the 10‐year follow‐up.

Mean ± SD	Major osteoporotic fractures		Vertebral fractures		Non‐vertebral fractures		Falls		Death	
	Yes (*n* = 260)	No (*n* = 760)	Yes (*n* = 174)	No (*n* = 820)	Yes (*n* = 107)	No (*n* = 1368)	Yes (*n* = 863)	No (*n* = 612)	Yes (*n* = 74)	No (*n* = 1401)
**Covariates**										
Age (years)	**66.8 ± 7.0**	**62.2 ± 6.7** **	**67.1 ± 6.9**	**62.3 ± 6.7** **	66.5 ± 7.4	64.4 ± 7.6*	64.6 ± 7.5	64.5 ± 7.7	**69.5 ± 7.2**	**64.3 ± 7.5** **
Height (cm)^a^	162.1 ± 7.0	161.9 ± 6.4	161.9 ± 6.9	162.0 ± 6.5	162.4 ± 7.5	161.2 ± 6.6	161.7 ± 6.6	160.7 ± 6.7	159.6 ± 6.8	161.4 ± 6.7*
BMI (kg/m^2^)	**26.2 ± 4.6**	**25.2 ± 4.2** **	26.2 ± 4.6	25.2 ± 4.2*	25.8 ± 4.3	25.9 ± 4.6	25.9 ± 4.5	26.0 ± 4.6*	26.8 ± 4.9	25.9 ± 4.5
Femoral neck BMD T‐score	**−1.3 ± 0.9**	**−1.0 ± 1.0** **	**−1.3 ± 1.0**	**−1.0 ± 1.0** **	**−1.4 ± 0.9**	**−1.1 ± 1.0** **	−1.1 ± 1.0	−1.1 ± 1.0	−1.3 ± 1.2	−1.1 ± 1.0*
Recent falls (% yes/no)	29.2 ± 45.6	25.8 ± 43.8	31.0 ± 46.4	26.0 ± 43.9	26.2 ± 44.2	25.3 ± 43.5	**31.7 ± 46.6**	**16.3 ± 37.0** **	16.2 ± 37.1	25.8 ± 43.8
Tobacco use (% yes/no)	18.5 ± 38.9	16.2 ± 36.9	17.8 ± 38.4	16.6 ± 37.2	18.7 ± 39.2	17.9 ± 38.4	16.7 ± 37.3	19.8 ± 39.9	21.6 ± 41.4	17.8 ± 38.2
Alcohol (% yes/no)	5.4 ± 22.6	4.6 ± 21.0	5.7 ± 23.3	4.8 ± 21.3	3.7 ± 19.1	4.8 ± 21.4	4.8 ± 21.3	4.7 ± 21.3	10.8 ± 31.3	4.4 ± 20.6*
Diabetes (% yes/no)	3.5 ± 18.3	2.5 ± 15.6	2.9 ± 16.8	2.6 ± 15.8	3.7 ± 19.1	4.1 ± 19.8	4.2 ± 20.0	3.9 ± 19.4	**12.2 ± 32.9**	**3.6 ± 18.7** **
Parental hip fracture (% yes/no)	13.2 ± 33.9	10.4 ± 30.6	13.4 ± 34.1	10.9 ± 31.2	15.1 ± 36.0	10.4 ± 30.6	11.9 ± 32.4	9.2 ± 28.9	9.5 ± 29.5	10.8 ± 31.1
Glucocorticoid use (% yes/no)	4.6 ± 21.0	3.0 ± 17.1	3.4 ± 18.3	3.2 ± 17.5	5.6 ± 23.1	3.3 ± 17.8	3.4 ± 18.0	3.6 ± 18.6	2.7 ± 16.3	3.5 ± 18.4
Polyarthritis (% yes/no)	1.9 ± 13.8	1.6 ± 12.5	2.3 ± 15.0	1.5 ± 12.0	1.9 ± 13.6	1.4 ± 11.7	1.5 ± 12.2	1.3 ± 11.4	1.4 ± 11.6	1.4 ± 11.9
FRAX MOF (% of 10‐year risk)	**15.4 ± 9.2**	**10.8 ± 6.3** **	**15.5 ± 8.8**	**11.0 ± 6.6** **	**15.7 ± 9.9**	**12.3 ± 7.6** **	12.8 ± 7.8	12.3 ± 7.8	**17.2 ± 10.7**	**12.3 ± 7.6** **
**Muscle parameters**										
HGS (kg.)^a^	**23.3 ± 6.1**	**24.8 ± 5.7** **	23.5 ± 5.9	24.8 ± 5.8*	23.1 ± 6.4	24.0 ± 5.9	23.9 ± 6.0	24.0 ± 5.8	22.7 ± 6.4	24.0 ± 5.9
ALM (kg.)	17.5 ± 2.9	17.1 ± 2.4	17.7 ± 3.0	17.1 ± 2.4	17.0 ± 2.6	17.1 ± 2.6	17.2 ± 2.6	16.9 ± 2.5	16.6 ± 2.8	17.1 ± 2.6
ALM/height^2^ (kg/m)	6.65 ± 0.93	6.5 ± 0.79	6.73 ± 0.98	6.49 ± 0.79*	6.46 ± 0.78	6.56 ± 0.85	6.56 ± 0.85	6.55 ± 0.84	6.56 ± 0.88	6.55 ± 0.84
ALM/BMI (^1^/m^2^)	0.676 ± 0.106	0.694 ± 0.102	0.680 ± 0.107	0.694 ± 0.103	0.674 ± 0.107	0.677 ± 0.103	0.682 ± 0.103	0.670 ± 0.105*	0.656 ± 0.111	0.678 ± 0.103

*Note:* All fractures: MOF defined by low‐trauma fracture at hip, humerus, forearm and vertebral; vertebral fractures: radiological Grades 2–3 or 2 Grade 1 fractures; non‐vertebral fracture: hip, humerus and forearm fractures; yes: event; no: no event; significant difference based on a p‐value for a two‐sided 95% confidence interval from Wilcoxon–Mann–Whitney test for continuous variables**.**

Abbreviations: ALM, appendicular lean mass; BMI, body mass index; SD, standard deviation.

^a^ two‐sided *t*‐test for normally distributed variable and chi‐squared test for dichotomous variables.

* p‐value under 0.05 were noted with.

** p‐value adjusted for Bonferroni under 0.0029 were considered as significant and were noted with **and bold.**

#### 2.6.1 Regression Analysis

Accelerated failure time (AFT) model analysis was used to assess the association of muscle strength or lean mass with the time to incident fragility fractures. AFT was chosen after a preliminary analysis with Cox proportional models, which failed the visual (scaled Schoenfeld residuals for each covariate) and numerical (proportional hazard test) assumptions. Logistic models are available in the Supporting Information. Note that a greater ratio from logistic model indicate a higher probability of event (higher risk), while a greater ratio from AFT indicate a longer time to event (lower risk). No dichotomous covariable was missing. Missing continuous covariables were replaced with the overall variable mean. All continuous covariables were normalized. The associations of interest were investigated using three logistic regression models: Model 1 (M1) was nonadjusted; M2 was adjusted for age, weight and height; Model 3 (M3) was additionally adjusted for recent falls, actual tobacco status, daily alcohol consumption over 3 units, diabetes, parental hip fracture, current glucocorticoid use, polyarthritis and femoral neck BMD T‐score. To avoid multicollinearity, the covariate weight was only included in the HGS model, and height was not included if the ALM index already included it (ALM/height^2^, ALM/(weight*height)). For each predictor and model, both AFT ratio and odds ratio (OR) were provided including their 95% CI. The C‐index and Area Under the Receiver Operator Curve (AUC) were calculated to evaluate the model's performance.

## 3 Results

**FIGURE 1:**
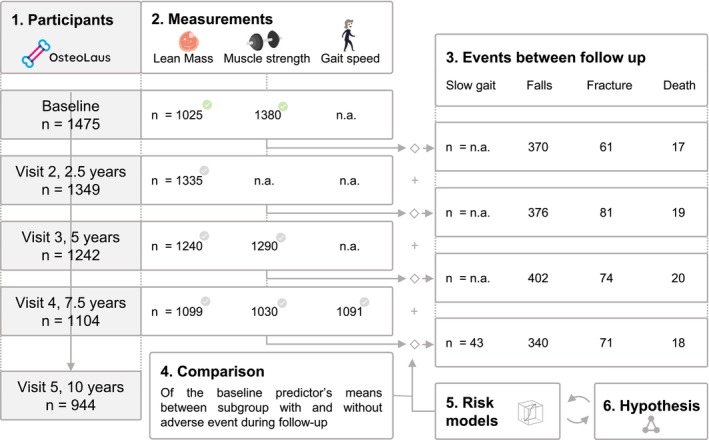
Participants, measurements, events and analysis flow chart. Main analysis with 10‐year follow‐up; supplementary analysis with shorter follow‐up; *measurements* were collected at each visits: lean mass by dual‐x‐ray absorptiometry; muscle strength from Jamar Hand dynamometer, gait speed from 6‐m walk test; each visit collected the *events* for the previous follow‐up: slow gait for new walking speed under 0.8 m/s, falls from questionnaire, fractures as one or more new fragility fracture and death.

This study included 1475 postmenopausal women from the baseline visit (age 64.5 ± 7.6 years, BMI 25.9 ± 4.5 kg/m^2^, ALM 17.1 ± 2.6 kg, HGS 23.9 ± 5.9 kg, Table 1). During the 10.2 ± 0.3 years of follow‐up, 74 women died, 863 had at least one fall, and 260 sustained at least one fragility fracture, including 174 vertebral, 66 forearm, 30 humerus and 21 hip fractures (Figure 1). At the end of follow‐up, 947 women remained (age 73.0 ± 6.9 years, BMI 25.7 ± 4.8 kg/m^2^, ALM 16.8 ± 2.5 kg, HGS 21.2 ± 5.5 kg), of whom 184 had a one or more fractures. During the last 2.5 years of follow‐up, 43 participants developed a slow gait speed. All participant's characteristics for each visit are described in Table 1. At baseline visit, 1025 women had available body composition data and 1380 HGS data. The availability of the predictors and the number of incident outcomes during each follow‐up are summarized in Figure 1.

### 3.1 Comparison of Baseline Characteristics

At baseline, participants with an incident MOF during the 10‐year follow‐up were 4.6 years older and had a 0.3 lower femoral neck BMD T‐score, and their FRAX was 4.6% higher (Table 2, *p* < 0.0029). Similar results were observed when comparing participants with and without VF, non‐VF and death, excepting age for non‐VF and femoral neck BMD T‐score for death, which were not significant. Patients with an incident MOF demonstrated a 1 kg/m^2^ greater BMI. Participants with falls during the 10‐year follow‐up demonstrated a 2.1 higher rate of previous falls at baseline. Participants who died during follow‐up demonstrated a 3.4 higher rate of diabetes at baseline. All other covariables were similar in the groups with or without fractures, falls or death.

Concerning muscle predictors, participants with an incident MOF had a 1.5‐kg lower HGS (−6.4%, Table 2) and a 0.9 lower HGS/arm lean mass (−7.8%, Table S1) at baseline compared to those without fracture. HGS measures were not different between women with VF and non‐VF compared with nonfractured women. There was no significant difference between ALM, ALM/height^2^ and ALM/BMI between fractured and nonfractured participants. The ALM indexes corrected for body weight were lower in participants with incident MOF versus without (Table S1: ALM/weight: −2.7%; and ALM/(height*weight): −3.1%, *p* < 0.0029). There was no significant difference between the participants with falls and death when comparing their baseline muscle characteristics (Table 2). Regarding MOF, falls and death outcomes, HGS and lean mass did not differ when considering shorter follow‐up durations of 5 and 2.5 years or when considering the different DXA device used (results not shown). In the last 2.5 years of follow‐up, 43 participants developed a slow gait speed, these had a 4.0 kg (−16.8%) lower HGS and 0.17 m/s (−15.0%) slower gait speed compared to the other (p < 0.0029).

A supplementary analysis included known Sarcopenia thresholds (Table S5). Participants with HGS under 20 kg (Sarcopenia Definitions and Outcomes Consortium ‐SDOC 2020) had a 1.79 (CI:1.29–2.49) greater odds for MOF and a 1.64 (CI: 1.12–2.39) greater odds for VF. Participants under the lean mass thresholds (ALM < 15 kg, ALM/height^2^ < 5.5 kg/m^2^, European Working Group on Sarcopenia in Older People—EWGSOP 2019) showed no association with any fracture group.

In a complementary analysis, we compared the association of lean mass with fracture based on two DXA devices (Hologic Discovery A or GE Lunar iDXA). The means of the different lean mass variables were not statistically different between each device and their 2.5‐ or 5‐year adverse events (results not shown).

### 3.2 Multivariable Modelling of Fragility Fractures

All AFT models (M1–3) are summarized in Table 3. In the multivariable analysis (M3), one SD increase in ALM (+2.58 kg) was associated with shorter time to event for MOF (AFT ratio: 0.72, CI: 0.61–0.85), VF (AFT ratio 0.67, CI: 0.55–0.82) but showed no association with non‐VF (AFT ratio 0.78, CI: 0.53–1.15). ALM/height^2^ provided similar results. One SD increase in ALM/BMI was associated with longer time to MOF (AFT ratio: 1.26, CI: 1.01–1.59) and non‐VF (AFT ratio: 1.98, CI: 1.21–3.25), but was not associated with VF. Similar results were demonstrated for total body lean mass and ALM (Table S3). The C‐index for the prediction of MOF (M3) was slightly greater when including ALM (0.708) compared to the C‐Index without ALM (0.684). Based on further lean mass parameters (Table S3), one SD increase in lean mass resulted in longer time to fracture if lean mass was corrected for weight (ALM/weight, ALM/(weight*height)) or regional mass (ALM, total body lean, arms lean or legs lean percent), and a shorter time to fracture if not corrected (total body, arms or legs lean mass).

**TABLE 3:** Prediction of 10‐year incident fragility fractures by grip strength (HGS) and appendicular lean mass (ALM) with accelerated failure time model.

Predictors and models		Major osteoporotic fractures (*n* = 260)		Vertebral fractures (G2–3 or 2xG1, *n* = 174)		Non‐vertebral fractures (*n* = 106)	
		AFT (95% CI)	C‐Index	AFT (95% CI)	C‐Index	AFT (95% CI)	C‐Index
M1: Univariate			—		—		—
M2: Age, BMD and height			0.679		0.688		0.706
M3: M2 + CRF			0.684		0.699		0.713
**HGS**	M1	**1.24 (1.10–1.40)**	0.595	**1.18 (1.03–1.36)**	0.574	**1.55 (1.18–2.05)**	0.619
	M2	1.09 (0.96–1.23)	0.694	1.03 (0.89–1.19)	0.704	**1.34 (1.01–1.76)**	0.722
	M3	1.10 (0.97–1.25)	0.699	1.03 (0.89–1.20)	0.710	**1.37 (1.03–1.81)**	0.732
**ALM**	M1	0.89 (0.77–1.03)	0.527	0.85 (0.72–1.00)	0.542	1.08 (0.79–1.50)	0.519
	M2	**0.72 (0.61–0.85)**	0.705	**0.68 (0.56–0.83)**	0.712	0.78 (0.53–1.13)	0.742
	M3	**0.72 (0.61–0.85)**	0.708	**0.67 (0.55–0.82)**	0.721	0.78 (0.53–1.15)	0.747
**ALM/height** ^**2**^	M1	0.87 (0.76–1.00)	0.534	**0.81 (0.69–0.95)**	0.556	1.07 (0.78–1.48)	0.521
	M2	**0.76 (0.66–0.88)**	0.704	**0.73 (0.61–0.86)**	0.711	0.80 (0.58–1.11)	0.741
	M3	**0.76 (0.66–0.88)**	0.708	**0.72 (0.60–0.85)**	0.720	0.81 (0.59–1.12)	0.747
**ALM/BMI**	M1	**1.18 (1.01–1.37)**	0.558	1.10 (0.93–1.31)	0.543	**1.49 (1.06–2.10)**	0.593
	M2	**1.29 (1.02–1.62)**	0.689	0.96 (0.74–1.26)	0.693	**2.06 (1.26–3.37)**	0.754
	M3	**1.26 (1.01–1.59)**	0.694	0.95 (0.73–1.24)	0.702	**1.98 (1.21–3.25)**	0.759

*Note:* AFT increase (AFT > 1.0) or decrease (AFT < 1.0) for 1 standard deviation increase in the predictor variable; significance level set for p‐value under 0.05 noted in **bold**; Model 1 (M1) as univariate regression; Model 2 (M2) including age, femoral neck BMD T‐score and height as covariates. HGS was additionally corrected for weight. M3 additionally including clinical risk factors (CRF): recent falls, actual tobacco status, daily alcohol consumption over 3 units, diabetes, parental hip fracture, current glucocorticoid use and polyarthritis; MOF defined by low‐trauma fracture at hip, humerus, forearm and vertebral; vertebral fractures: radiological (accelerated failure time model with Weibull distribution) the ratio represent time‐to‐event Grades 2–3 or 2 Grade 1 fractures; non‐vertebral fracture: hip, humerus and forearm fractures.

Abbreviations: ALM, appendicular lean mass; BMI, body mass index; CI, confidence interval; HGS, handgrip strength.

One SD increase in HGS (+5.92 kg) was associated with longer time to event for MOF (AFT: 1.24, 95%CI: 1.10–1.40, *p* < 0.05), VF (AFT: 1.18, CI: 1.05–1.36) and non‐VF (AFT: 1.55, CI: 1.18–1.2.05) in the univariate analysis. In the multivariable models (M2 and M3), one SD increase in HGS was only associated with longer time to event for non‐VF (M3: AFT 1.37, CI: 1.03–1.81).

Additional logistic model demonstrated similar trend (Table S4). When including the non‐VF sites separately, no muscle parameters demonstrated a significant association with fractures (results not shown). There was also no difference in the main results when excluding the MOF defined by the occurrence of two Grade 1 VF (*n* = 6). There was also no difference in the main results when considering both HGS and ALM as covariables. An additional model adjusted only for the FRAX 10‐year risk of MOF yielded lower performance (AUC) than M3 and was thus not shown.

## 4 Discussion

This 10‐year prospective and population‐based study of 1475 postmenopausal women highlights how the muscle parameters are associated with osteoporotic fractures.

### 4.1 Lean Mass and Prediction of Fragility Fractures

The main result is that lean mass and its indexes were independently associated with incident fragility fractures. In the multivariable model, one SD increase in ALM/BMI was associated with a 26% longer time to MOF, while ALM without correction for body weight was associated with a 28% shorter time to MOF. To the best of our knowledge, our study is the first to measure lean mass with DXA and report shorter time to VF (AFT model) or higher odds of VF (logistic model), by increase in ALM and ALM/height^2^ [10]. In 1281 Korean women of 71.0 ± 4.4 years, Lee et al. found no association between lean mass measured with Body Impedance Analysis (BIA) and 12‐year risk of VF [18]. Hong et al. found a lower 3‐year risk of VF (OR 0.55, CI 0.43–0.71; highest vs. lowest quartile) in 158′426 Korean men but no difference in 131′587 women, using the Lee Equation estimation of lean mass [19]. These two previous studies on VF included Asian population, which is known to differ in body composition, and they measured lean mass with BIA and an estimation equation, respectively.

In the supplementary analysis, our study demonstrated no discrimination advantage between the two DXA devices tested; however, these results are limited by the different dates and follow‐ups used for these assessments.

### 4.2 Importance of Fat Mass and Weight in Fragility Fracture Prediction

The second main result is the lean mass interdependence with weight and fat in the prediction of incident MOF, VF or non‐VF. When analysing VF and non‐VF separately in the AFT models, ALM and ALM/height^2^ were only significant with VF, while ALM/BMI, ALM/weight, ALM/(weight*height) and ALM percent were only significant for non‐VF. Similar trends were demonstrated in the logistic models, where lean mass alone was positively associated with MOF and VF, but lean mass corrected or stratified by body weight or fat, respectively, was not associated. The VF, BMI, body fat [20] and visceral adipose tissue [21] were already associated with prevalent vertebral deformities. This relation of weight with fragility fractures is subject to many hypotheses including ethnical (e.g. variation of BMI and body fat), mechanical (site‐specific mechanism, BMD adaptation and soft tissue cushioning) or endocrine hypotheses (oestrogen, insulin, vitamin D and cytokines) [22, 23, 24]. VF can occur spontaneously [25], while non‐VF mostly follows a fall or a minimal trauma [3]. As total body lean mass represents 60% of the total mass (Table S2), statistical approaches, as demonstrated in our study, need to consider this collinearity by adjusting lean mass by weight (total mass) or fat mass (rest of tissues mass without bone mass), by including it as a covariate in the multivariable model or using stratification.

Most of previous studies on MOF only accounted for weight indirectly through the FRAX value [26, 27, 28, 29, 30, 31]. This weight dependent trend was only described in one Chinese cohort (MrOs China), where one SD increase in ALM/height was a protective factor and ALM/weight was a risk factor for MOF [27]. These finding suggesting the opposite might be explained by the inclusion of the symptomatic VF only. With more than 70% of VF being asymptomatic [25], this study underestimates the proportion of VF, and the results might be driven by non‐VF. Further studies are needed to clarify this fracture site dependance, while considering the complex collinearity between lean mass and weight and considering the ethnical or sex differences.

### 4.3 Muscle Strength and Prediction of Fragility Fractures

HGS and its muscle‐specific strength (HGS/arm lean mass) were both significantly lower at baseline in the individuals with incident MOF. In the multivariable model, one SD increase in HGS was associated with a 24% longer time to MOF. Muscle strength can be described as a protective factor for fragility fractures.

Only two previous studies in 1518 Chinese women [27] and 1342 Japanese women [32] demonstrated higher HGS as a protective factor for MOF. In men, it was demonstrated as a protective factor in six previous studies [26, 27, 29, 30, 33, 34]. HGS/arm lean mass was reported as a protective factor in the analysis of 9512 men by Cawthon et al. using a Cox proportional model [35]. The concept of muscle‐specific strength is a promising marker of muscle health, as this might better depict the multifactorial components of muscle health [36]. More studies are needed, including larger sample size and other muscle groups than forearm muscles.

### 4.4 Modelling Incident Fragility Fractures With Muscle Parameters

**FIGURE 2:**
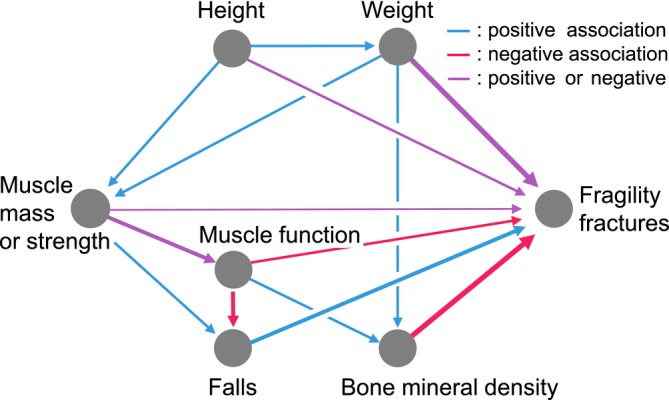
Directed acyclic graph: hypothesis from muscle parameters to incident fragility fractures. Directed acyclic graph (DAG) including the association importance (width), the excepted positive or negative association (colour) and the expected direction of association based on previous studies and the OsteoLaus cohort; this graphic does not include other important risk factors such as age, current smoking status, daily alcohol consumption over 3 units and history of diabetes; these hypothesis were tested in the multivariate models and vary between the types of fragility fractures (see Table 3 and Table S3).

Muscle parameters and fragility fractures are influenced by multiple parameters and are interconnected (see methodology and Figure 2). From a clinical perspective, these findings suggest only a small improvement of fracture prediction by muscle parameters compared to previous models mostly developed for hip or MOF prediction (Tables 3 and Table S3) [37]. Regardless limited statistical power of the current analysis, we notice a consistent trend of the muscle parameters association differing between VF and non‐VF. This suggests a site‐specific and/or bone type‐specific nature of the fracture prediction ability of muscle parameters. Further investigation of muscle parameters prediction of specific fracture types would help to understand the underlying mechanical and physiological mechanisms. As known, antiosteoporotic treatments' effect differs among the fractures' types [4]. Hence, better understanding the fracture‐specific prediction ability of muscle parameters would eventually improve treatment choice. However, the acquisition of a total body DXA scan and the body composition analysis has its cost and its clinical time. Its inclusion in clinical routine should be evaluated from the added value in bone fragility prediction and the cost‐effectiveness point of view. Nevertheless, this exam remains of greater interest to study the muscle physiopathology including its related diseases such as sarcopenia, cachexia and malnutrition [8].

### 4.5 Association of Muscle Parameters With Falls, Slow Gait Speed and Mortality

Lower muscle strength (HGS) and slower gait speed were seen in the participants with incident slow gait speed. However, muscle parameters were not different in the participants with falls and death, while many previous studies had demonstrated such association [9, 35, 38]. As these secondary outcomes are exponentially increasing with ageing, their association might be mitigated by the relatively young and healthy sample studied in the OsteoLaus cohort, limiting our statistical power.

### 4.6 Strengths and Limitations

The main strength of this study is the observational and population‐based design, the results are more representative to the general population, allowing inference in broad contexts. Second, the relative long follow‐up duration of 10‐years resulted in a high number of incident adverse events increasing the statistical power. Similarly, this prospective design leads to more evidence for an eventual causal association as the exposures (muscle parameters) precede the adverse events (falls, fractures and death). Third, all women underwent lateral spine DXA imaging, which is the most common for VF screening, as more than 70% of fractures are asymptomatic [25].

This study has several limitations. First, the interpretation of its results is limited to women only, regardless of the known variation of muscle parameters between genders [10]. Second, we considered only linear relations between the covariable and fragility fractures, based on visual regression plots with first‐, second‐ and third‐order equations, while U‐shaped association were previously described [23]. Stratification for fat and weight was thus performed. Third, this study did not compare the adverse event between the sarcopenia definitions due to the limited number of events in each sarcopenia subgroups. By comparing the parameters separately, this study provides insight on the parameters independently of the definition used. Fourth, even if DXA is precise and reliable, its assessment of lean mass also includes water, joints and ligaments [39]. Further studies on muscle mass could also consider magnetic resonance imaging, computed tomography, creatine dilution test (D3‐creatine), BIA and ultrasound based on their study setting [40].

## 5 Conclusion

This study highlights the importance of muscle health indicators in predicting fragility fractures among postmenopausal women. Over a 10‐year follow‐up, muscle strength and lean mass indices were independently associated with fragility fractures. Baseline muscle parameters were not different for participant with or without incident fall or death. Multivariable models showed that both grip strength and lean mass were independently associated with incident fractures. A careful consideration of body weight and fat mass is needed. These parameters only slightly improved the prediction model performance. Future research including larger sample sizes, state‐of‐the‐art muscle health assessment, other muscle groups and muscle‐specific strength are needed to better understand the pathophysiology of muscle health and fractures. These efforts are needed to close the gap in effective prediction and management of individuals at high risk for fragility fractures, ultimately reducing the burden of fragility fractures on ageing populations.

## Conflicts of Interest

The authors declare no conflicts of interest.

## Supporting information


**Table S1.** Comparison of baseline lean mass assessments between participants with or without fragility fractures in the 10‐year follow‐up.
**Table S2** Comparison of baseline muscle strength and lean mass assessments between participants with or without hip, humerus or forearm fractures in the 10‐year follow‐up.
**Table S3.** Prediction of 10‐year incident fragility fractures by lean mass with accelerated failure time model.
**Table S4.** Prediction of 10‐year incident fragility fractures by handgrip strength (HGS) and appendicular lean mass (ALM) with multivariable logistic regression.
**Table S5.** 10‐year incident fragility fractures odds based on sarcopenia thresholds for handgrip strength (HGS) and appendicular lean mass (ALM).
