# Supplementary material for: Prediction of 10‐Year Fragility Fractures Using Muscle Health Indicators in Postmenopausal Women: The OsteoLaus Cohort
Source: J Cachexia Sarcopenia Muscle. 2025 Jun 4;16(3):e13837. doi: 10.1002/jcsm.13837 (PMC12134769; doi:10.1002/jcsm.13837)
Supplement: Supplementary file 1 — Table S1. Comparison of baseline lean mass assessments between participants with or without fragility fractures in the 10‐year follow‐up. Table S2 Comparison of baseline muscle strength and lean mass assessments between participants with or without hip, humerus or forearm fractures in the 10‐year follow‐up. Table S3. Prediction of 10‐year incident fragility fractures by lean mass with accelerated failure time model. Table S4. Prediction of 10‐year incident fragility fractures by handgrip strength (HGS) and appendicular lean mass (ALM) with multivariable logistic regression. Table S5. 10‐year incident fragility fractures odds based on sarcopenia thresholds for handgrip strength (HGS) and appendicular lean mass (ALM). [file JCSM-16-e13837-s001.pptx]

## Slide 1
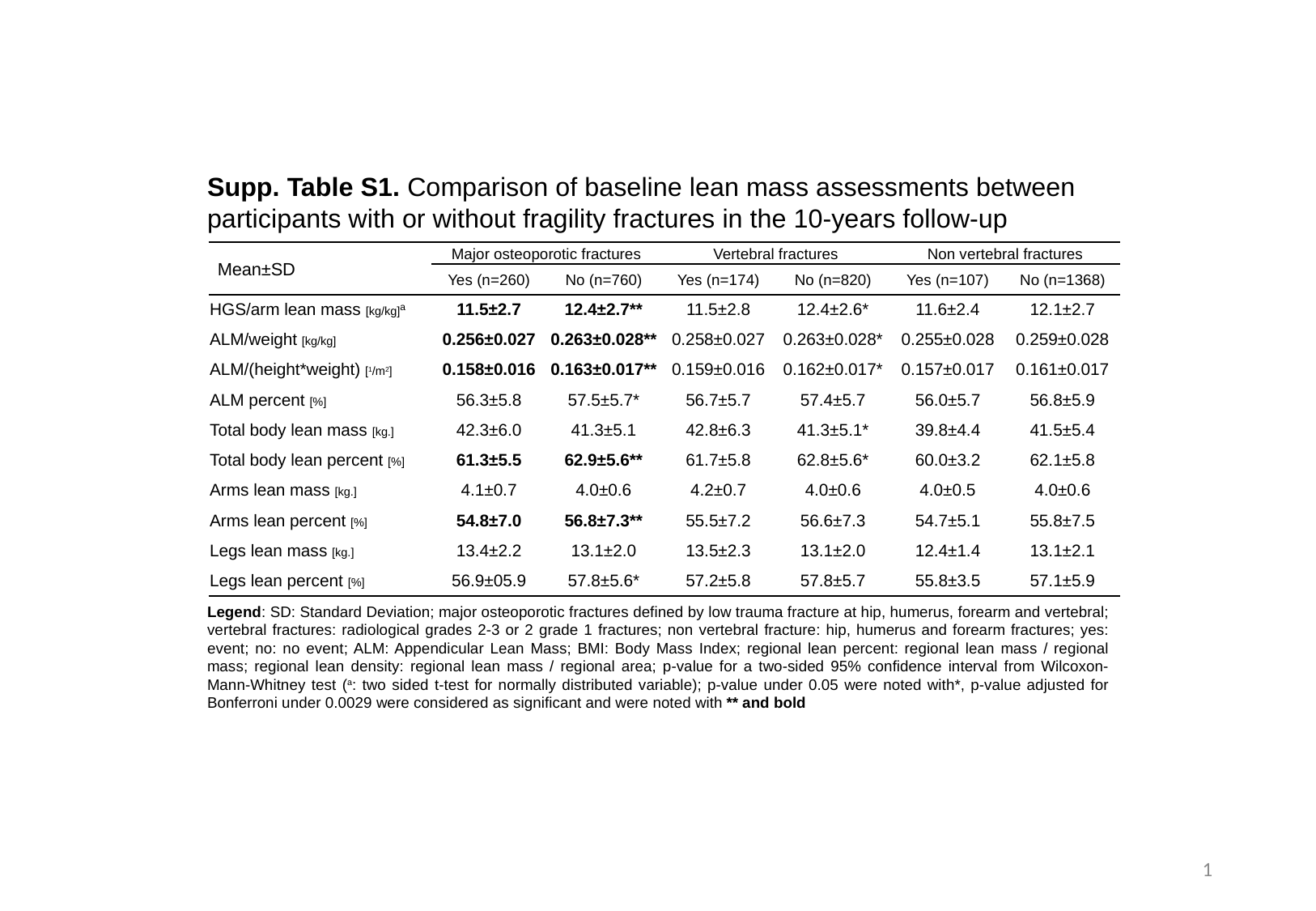

Supp. Table S1. Comparison of baseline lean mass assessments between participants with or without fragility fractures in the 10-years follow-up
| Mean±SD | Major osteoporotic fractures | | Vertebral fractures | | Non vertebral fractures | |
| --- | --- | --- | --- | --- | --- | --- |
| | Yes (n=260) | No (n=760) | Yes (n=174) | No (n=820) | Yes (n=107) | No (n=1368) |
| HGS/arm lean mass [kg/kg]a | 11.5±2.7 | 12.4±2.7\*\* | 11.5±2.8 | 12.4±2.6\* | 11.6±2.4 | 12.1±2.7 |
| ALM/weight [kg/kg] | 0.256±0.027 | 0.263±0.028\*\* | 0.258±0.027 | 0.263±0.028\* | 0.255±0.028 | 0.259±0.028 |
| ALM/(height\*weight) [1/m2] | 0.158±0.016 | 0.163±0.017\*\* | 0.159±0.016 | 0.162±0.017\* | 0.157±0.017 | 0.161±0.017 |
| ALM percent [%] | 56.3±5.8 | 57.5±5.7\* | 56.7±5.7 | 57.4±5.7 | 56.0±5.7 | 56.8±5.9 |
| Total body lean mass [kg.] | 42.3±6.0 | 41.3±5.1 | 42.8±6.3 | 41.3±5.1\* | 39.8±4.4 | 41.5±5.4 |
| Total body lean percent [%] | 61.3±5.5 | 62.9±5.6\*\* | 61.7±5.8 | 62.8±5.6\* | 60.0±3.2 | 62.1±5.8 |
| Arms lean mass [kg.] | 4.1±0.7 | 4.0±0.6 | 4.2±0.7 | 4.0±0.6 | 4.0±0.5 | 4.0±0.6 |
| Arms lean percent [%] | 54.8±7.0 | 56.8±7.3\*\* | 55.5±7.2 | 56.6±7.3 | 54.7±5.1 | 55.8±7.5 |
| Legs lean mass [kg.] | 13.4±2.2 | 13.1±2.0 | 13.5±2.3 | 13.1±2.0 | 12.4±1.4 | 13.1±2.1 |
| Legs lean percent [%] | 56.9±05.9 | 57.8±5.6\* | 57.2±5.8 | 57.8±5.7 | 55.8±3.5 | 57.1±5.9 |
Legend: SD: Standard Deviation; major osteoporotic fractures defined by low trauma fracture at hip, humerus, forearm and vertebral; vertebral fractures: radiological grades 2-3 or 2 grade 1 fractures; non vertebral fracture: hip, humerus and forearm fractures; yes: event; no: no event; ALM: Appendicular Lean Mass; BMI: Body Mass Index; regional lean percent: regional lean mass / regional mass; regional lean density: regional lean mass / regional area; p-value for a two-sided 95% confidence interval from Wilcoxon-Mann-Whitney test (a: two sided t-test for normally distributed variable); p-value under 0.05 were noted with*, p-value adjusted for Bonferroni under 0.0029 were considered as significant and were noted with ** and bold
1

## Slide 2
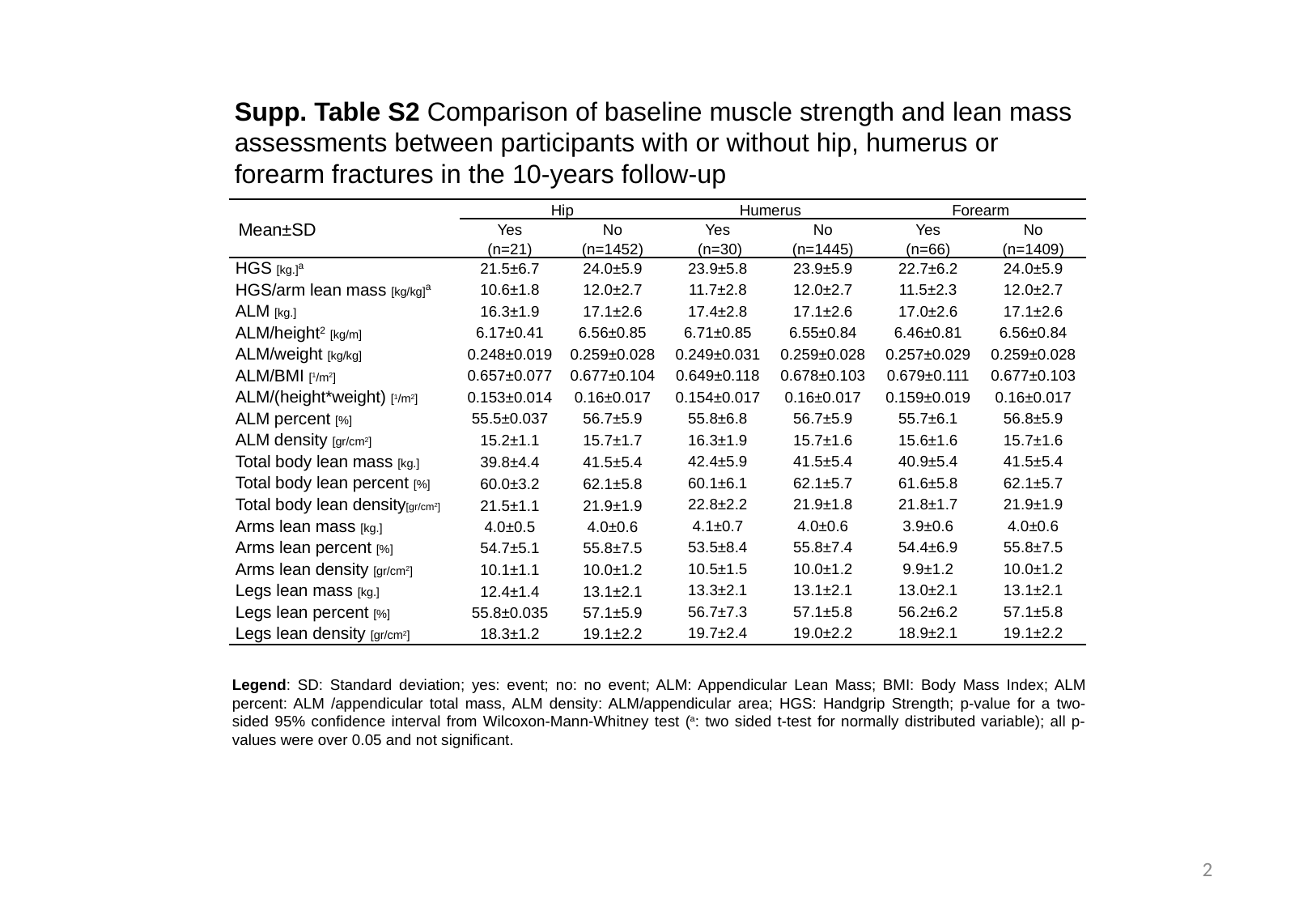

Supp. Table S2 Comparison of baseline muscle strength and lean mass assessments between participants with or without hip, humerus or forearm fractures in the 10-years follow-up
| Mean±SD | Hip | | Humerus | | Forearm | |
| --- | --- | --- | --- | --- | --- | --- |
| | Yes (n=21) | No (n=1452) | Yes (n=30) | No (n=1445) | Yes (n=66) | No (n=1409) |
| HGS [kg.]a | 21.5±6.7 | 24.0±5.9 | 23.9±5.8 | 23.9±5.9 | 22.7±6.2 | 24.0±5.9 |
| HGS/arm lean mass [kg/kg]a | 10.6±1.8 | 12.0±2.7 | 11.7±2.8 | 12.0±2.7 | 11.5±2.3 | 12.0±2.7 |
| ALM [kg.] | 16.3±1.9 | 17.1±2.6 | 17.4±2.8 | 17.1±2.6 | 17.0±2.6 | 17.1±2.6 |
| ALM/height2 [kg/m] | 6.17±0.41 | 6.56±0.85 | 6.71±0.85 | 6.55±0.84 | 6.46±0.81 | 6.56±0.84 |
| ALM/weight [kg/kg] | 0.248±0.019 | 0.259±0.028 | 0.249±0.031 | 0.259±0.028 | 0.257±0.029 | 0.259±0.028 |
| ALM/BMI [1/m2] | 0.657±0.077 | 0.677±0.104 | 0.649±0.118 | 0.678±0.103 | 0.679±0.111 | 0.677±0.103 |
| ALM/(height\*weight) [1/m2] | 0.153±0.014 | 0.16±0.017 | 0.154±0.017 | 0.16±0.017 | 0.159±0.019 | 0.16±0.017 |
| ALM percent [%] | 55.5±0.037 | 56.7±5.9 | 55.8±6.8 | 56.7±5.9 | 55.7±6.1 | 56.8±5.9 |
| ALM density [gr/cm2] | 15.2±1.1 | 15.7±1.7 | 16.3±1.9 | 15.7±1.6 | 15.6±1.6 | 15.7±1.6 |
| Total body lean mass [kg.] | 39.8±4.4 | 41.5±5.4 | 42.4±5.9 | 41.5±5.4 | 40.9±5.4 | 41.5±5.4 |
| Total body lean percent [%] | 60.0±3.2 | 62.1±5.8 | 60.1±6.1 | 62.1±5.7 | 61.6±5.8 | 62.1±5.7 |
| Total body lean density[gr/cm2] | 21.5±1.1 | 21.9±1.9 | 22.8±2.2 | 21.9±1.8 | 21.8±1.7 | 21.9±1.9 |
| Arms lean mass [kg.] | 4.0±0.5 | 4.0±0.6 | 4.1±0.7 | 4.0±0.6 | 3.9±0.6 | 4.0±0.6 |
| Arms lean percent [%] | 54.7±5.1 | 55.8±7.5 | 53.5±8.4 | 55.8±7.4 | 54.4±6.9 | 55.8±7.5 |
| Arms lean density [gr/cm2] | 10.1±1.1 | 10.0±1.2 | 10.5±1.5 | 10.0±1.2 | 9.9±1.2 | 10.0±1.2 |
| Legs lean mass [kg.] | 12.4±1.4 | 13.1±2.1 | 13.3±2.1 | 13.1±2.1 | 13.0±2.1 | 13.1±2.1 |
| Legs lean percent [%] | 55.8±0.035 | 57.1±5.9 | 56.7±7.3 | 57.1±5.8 | 56.2±6.2 | 57.1±5.8 |
| Legs lean density [gr/cm2] | 18.3±1.2 | 19.1±2.2 | 19.7±2.4 | 19.0±2.2 | 18.9±2.1 | 19.1±2.2 |
Legend: SD: Standard deviation; yes: event; no: no event; ALM: Appendicular Lean Mass; BMI: Body Mass Index; ALM percent: ALM /appendicular total mass, ALM density: ALM/appendicular area; HGS: Handgrip Strength; p-value for a two-sided 95% confidence interval from Wilcoxon-Mann-Whitney test (a: two sided t-test for normally distributed variable); all p-values were over 0.05 and not significant.
2

## Slide 3
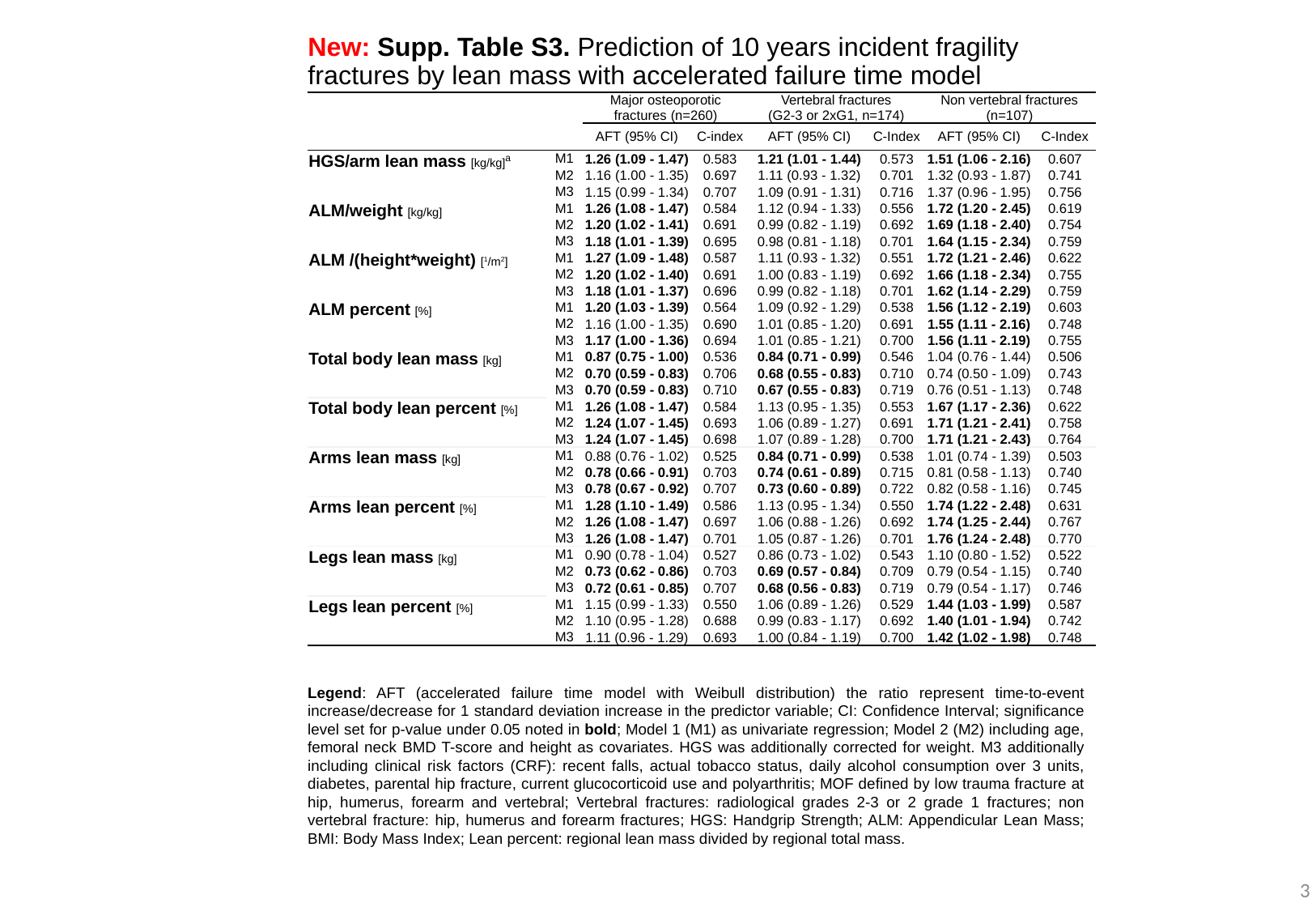

# New: Supp. Table S3. Prediction of 10 years incident fragility fractures by lean mass with accelerated failure time model
| | | Major osteoporotic fractures (n=260) | | Vertebral fractures (G2-3 or 2xG1, n=174) | | Non vertebral fractures (n=107) | |
| --- | --- | --- | --- | --- | --- | --- | --- |
| | | AFT (95% CI) | C-index | AFT (95% CI) | C-Index | AFT (95% CI) | C-Index |
| HGS/arm lean mass [kg/kg]a | M1 | 1.26 (1.09 - 1.47) | 0.583 | 1.21 (1.01 - 1.44) | 0.573 | 1.51 (1.06 - 2.16) | 0.607 |
| | M2 | 1.16 (1.00 - 1.35) | 0.697 | 1.11 (0.93 - 1.32) | 0.701 | 1.32 (0.93 - 1.87) | 0.741 |
| | M3 | 1.15 (0.99 - 1.34) | 0.707 | 1.09 (0.91 - 1.31) | 0.716 | 1.37 (0.96 - 1.95) | 0.756 |
| ALM/weight [kg/kg] | M1 | 1.26 (1.08 - 1.47) | 0.584 | 1.12 (0.94 - 1.33) | 0.556 | 1.72 (1.20 - 2.45) | 0.619 |
| | M2 | 1.20 (1.02 - 1.41) | 0.691 | 0.99 (0.82 - 1.19) | 0.692 | 1.69 (1.18 - 2.40) | 0.754 |
| | M3 | 1.18 (1.01 - 1.39) | 0.695 | 0.98 (0.81 - 1.18) | 0.701 | 1.64 (1.15 - 2.34) | 0.759 |
| ALM /(height\*weight) [1/m2] | M1 | 1.27 (1.09 - 1.48) | 0.587 | 1.11 (0.93 - 1.32) | 0.551 | 1.72 (1.21 - 2.46) | 0.622 |
| | M2 | 1.20 (1.02 - 1.40) | 0.691 | 1.00 (0.83 - 1.19) | 0.692 | 1.66 (1.18 - 2.34) | 0.755 |
| | M3 | 1.18 (1.01 - 1.37) | 0.696 | 0.99 (0.82 - 1.18) | 0.701 | 1.62 (1.14 - 2.29) | 0.759 |
| ALM percent [%] | M1 | 1.20 (1.03 - 1.39) | 0.564 | 1.09 (0.92 - 1.29) | 0.538 | 1.56 (1.12 - 2.19) | 0.603 |
| | M2 | 1.16 (1.00 - 1.35) | 0.690 | 1.01 (0.85 - 1.20) | 0.691 | 1.55 (1.11 - 2.16) | 0.748 |
| | M3 | 1.17 (1.00 - 1.36) | 0.694 | 1.01 (0.85 - 1.21) | 0.700 | 1.56 (1.11 - 2.19) | 0.755 |
| Total body lean mass [kg] | M1 | 0.87 (0.75 - 1.00) | 0.536 | 0.84 (0.71 - 0.99) | 0.546 | 1.04 (0.76 - 1.44) | 0.506 |
| | M2 | 0.70 (0.59 - 0.83) | 0.706 | 0.68 (0.55 - 0.83) | 0.710 | 0.74 (0.50 - 1.09) | 0.743 |
| | M3 | 0.70 (0.59 - 0.83) | 0.710 | 0.67 (0.55 - 0.83) | 0.719 | 0.76 (0.51 - 1.13) | 0.748 |
| Total body lean percent [%] | M1 | 1.26 (1.08 - 1.47) | 0.584 | 1.13 (0.95 - 1.35) | 0.553 | 1.67 (1.17 - 2.36) | 0.622 |
| | M2 | 1.24 (1.07 - 1.45) | 0.693 | 1.06 (0.89 - 1.27) | 0.691 | 1.71 (1.21 - 2.41) | 0.758 |
| | M3 | 1.24 (1.07 - 1.45) | 0.698 | 1.07 (0.89 - 1.28) | 0.700 | 1.71 (1.21 - 2.43) | 0.764 |
| Arms lean mass [kg] | M1 | 0.88 (0.76 - 1.02) | 0.525 | 0.84 (0.71 - 0.99) | 0.538 | 1.01 (0.74 - 1.39) | 0.503 |
| | M2 | 0.78 (0.66 - 0.91) | 0.703 | 0.74 (0.61 - 0.89) | 0.715 | 0.81 (0.58 - 1.13) | 0.740 |
| | M3 | 0.78 (0.67 - 0.92) | 0.707 | 0.73 (0.60 - 0.89) | 0.722 | 0.82 (0.58 - 1.16) | 0.745 |
| Arms lean percent [%] | M1 | 1.28 (1.10 - 1.49) | 0.586 | 1.13 (0.95 - 1.34) | 0.550 | 1.74 (1.22 - 2.48) | 0.631 |
| | M2 | 1.26 (1.08 - 1.47) | 0.697 | 1.06 (0.88 - 1.26) | 0.692 | 1.74 (1.25 - 2.44) | 0.767 |
| | M3 | 1.26 (1.08 - 1.47) | 0.701 | 1.05 (0.87 - 1.26) | 0.701 | 1.76 (1.24 - 2.48) | 0.770 |
| Legs lean mass [kg] | M1 | 0.90 (0.78 - 1.04) | 0.527 | 0.86 (0.73 - 1.02) | 0.543 | 1.10 (0.80 - 1.52) | 0.522 |
| | M2 | 0.73 (0.62 - 0.86) | 0.703 | 0.69 (0.57 - 0.84) | 0.709 | 0.79 (0.54 - 1.15) | 0.740 |
| | M3 | 0.72 (0.61 - 0.85) | 0.707 | 0.68 (0.56 - 0.83) | 0.719 | 0.79 (0.54 - 1.17) | 0.746 |
| Legs lean percent [%] | M1 | 1.15 (0.99 - 1.33) | 0.550 | 1.06 (0.89 - 1.26) | 0.529 | 1.44 (1.03 - 1.99) | 0.587 |
| | M2 | 1.10 (0.95 - 1.28) | 0.688 | 0.99 (0.83 - 1.17) | 0.692 | 1.40 (1.01 - 1.94) | 0.742 |
| | M3 | 1.11 (0.96 - 1.29) | 0.693 | 1.00 (0.84 - 1.19) | 0.700 | 1.42 (1.02 - 1.98) | 0.748 |
Legend: AFT (accelerated failure time model with Weibull distribution) the ratio represent time-to-event increase/decrease for 1 standard deviation increase in the predictor variable; CI: Confidence Interval; significance level set for p-value under 0.05 noted in bold; Model 1 (M1) as univariate regression; Model 2 (M2) including age, femoral neck BMD T-score and height as covariates. HGS was additionally corrected for weight. M3 additionally including clinical risk factors (CRF): recent falls, actual tobacco status, daily alcohol consumption over 3 units, diabetes, parental hip fracture, current glucocorticoid use and polyarthritis; MOF defined by low trauma fracture at hip, humerus, forearm and vertebral; Vertebral fractures: radiological grades 2-3 or 2 grade 1 fractures; non vertebral fracture: hip, humerus and forearm fractures; HGS: Handgrip Strength; ALM: Appendicular Lean Mass; BMI: Body Mass Index; Lean percent: regional lean mass divided by regional total mass.
3

## Slide 4
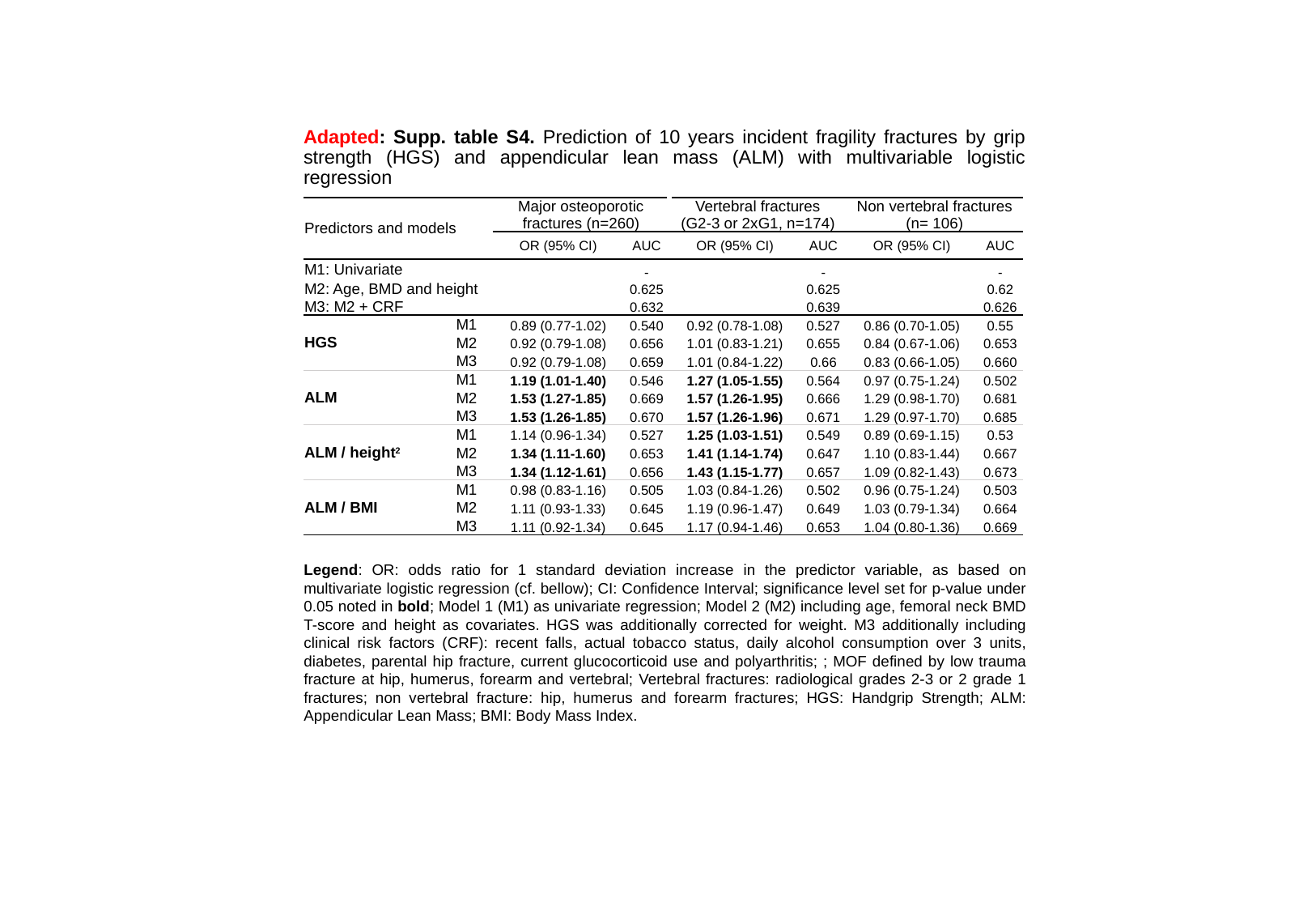

# Adapted: Supp. table S4. Prediction of 10 years incident fragility fractures by grip strength (HGS) and appendicular lean mass (ALM) with multivariable logistic regression
| Predictors and models | | Major osteoporotic fractures (n=260) | | Vertebral fractures (G2-3 or 2xG1, n=174) | | Non vertebral fractures (n= 106) | |
| --- | --- | --- | --- | --- | --- | --- | --- |
| | | OR (95% CI) | AUC | OR (95% CI) | AUC | OR (95% CI) | AUC |
| M1: Univariate | | | - | | - | | - |
| M2: Age, BMD and height | | | 0.625 | | 0.625 | | 0.62 |
| M3: M2 + CRF | | | 0.632 | | 0.639 | | 0.626 |
| HGS | M1 | 0.89 (0.77-1.02) | 0.540 | 0.92 (0.78-1.08) | 0.527 | 0.86 (0.70-1.05) | 0.55 |
| | M2 | 0.92 (0.79-1.08) | 0.656 | 1.01 (0.83-1.21) | 0.655 | 0.84 (0.67-1.06) | 0.653 |
| | M3 | 0.92 (0.79-1.08) | 0.659 | 1.01 (0.84-1.22) | 0.66 | 0.83 (0.66-1.05) | 0.660 |
| ALM | M1 | 1.19 (1.01-1.40) | 0.546 | 1.27 (1.05-1.55) | 0.564 | 0.97 (0.75-1.24) | 0.502 |
| | M2 | 1.53 (1.27-1.85) | 0.669 | 1.57 (1.26-1.95) | 0.666 | 1.29 (0.98-1.70) | 0.681 |
| | M3 | 1.53 (1.26-1.85) | 0.670 | 1.57 (1.26-1.96) | 0.671 | 1.29 (0.97-1.70) | 0.685 |
| ALM / height2 | M1 | 1.14 (0.96-1.34) | 0.527 | 1.25 (1.03-1.51) | 0.549 | 0.89 (0.69-1.15) | 0.53 |
| | M2 | 1.34 (1.11-1.60) | 0.653 | 1.41 (1.14-1.74) | 0.647 | 1.10 (0.83-1.44) | 0.667 |
| | M3 | 1.34 (1.12-1.61) | 0.656 | 1.43 (1.15-1.77) | 0.657 | 1.09 (0.82-1.43) | 0.673 |
| ALM / BMI | M1 | 0.98 (0.83-1.16) | 0.505 | 1.03 (0.84-1.26) | 0.502 | 0.96 (0.75-1.24) | 0.503 |
| | M2 | 1.11 (0.93-1.33) | 0.645 | 1.19 (0.96-1.47) | 0.649 | 1.03 (0.79-1.34) | 0.664 |
| | M3 | 1.11 (0.92-1.34) | 0.645 | 1.17 (0.94-1.46) | 0.653 | 1.04 (0.80-1.36) | 0.669 |
Legend: OR: odds ratio for 1 standard deviation increase in the predictor variable, as based on multivariate logistic regression (cf. bellow); CI: Confidence Interval; significance level set for p-value under 0.05 noted in bold; Model 1 (M1) as univariate regression; Model 2 (M2) including age, femoral neck BMD T-score and height as covariates. HGS was additionally corrected for weight. M3 additionally including clinical risk factors (CRF): recent falls, actual tobacco status, daily alcohol consumption over 3 units, diabetes, parental hip fracture, current glucocorticoid use and polyarthritis; ; MOF defined by low trauma fracture at hip, humerus, forearm and vertebral; Vertebral fractures: radiological grades 2-3 or 2 grade 1 fractures; non vertebral fracture: hip, humerus and forearm fractures; HGS: Handgrip Strength; ALM: Appendicular Lean Mass; BMI: Body Mass Index.

## Slide 5
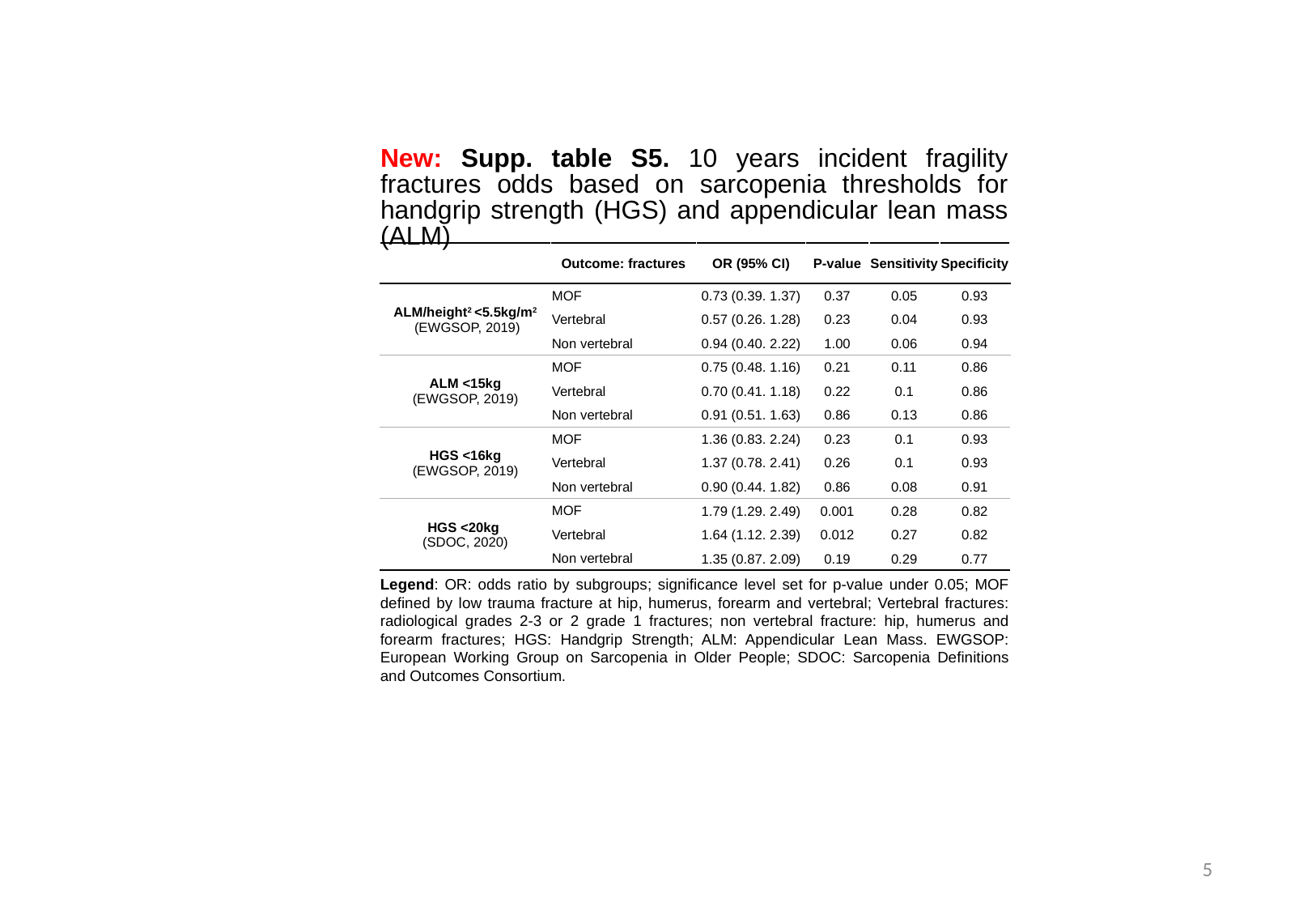

# New: Supp. table S5. 10 years incident fragility fractures odds based on sarcopenia thresholds for handgrip strength (HGS) and appendicular lean mass (ALM)
| | Outcome: fractures | OR (95% CI) | P-value | Sensitivity | Specificity |
| --- | --- | --- | --- | --- | --- |
| ALM/height2 <5.5kg/m2 (EWGSOP, 2019) | MOF | 0.73 (0.39. 1.37) | 0.37 | 0.05 | 0.93 |
| | Vertebral | 0.57 (0.26. 1.28) | 0.23 | 0.04 | 0.93 |
| | Non vertebral | 0.94 (0.40. 2.22) | 1.00 | 0.06 | 0.94 |
| ALM <15kg (EWGSOP, 2019) | MOF | 0.75 (0.48. 1.16) | 0.21 | 0.11 | 0.86 |
| | Vertebral | 0.70 (0.41. 1.18) | 0.22 | 0.1 | 0.86 |
| | Non vertebral | 0.91 (0.51. 1.63) | 0.86 | 0.13 | 0.86 |
| HGS <16kg (EWGSOP, 2019) | MOF | 1.36 (0.83. 2.24) | 0.23 | 0.1 | 0.93 |
| | Vertebral | 1.37 (0.78. 2.41) | 0.26 | 0.1 | 0.93 |
| | Non vertebral | 0.90 (0.44. 1.82) | 0.86 | 0.08 | 0.91 |
| HGS <20kg (SDOC, 2020) | MOF | 1.79 (1.29. 2.49) | 0.001 | 0.28 | 0.82 |
| | Vertebral | 1.64 (1.12. 2.39) | 0.012 | 0.27 | 0.82 |
| | Non vertebral | 1.35 (0.87. 2.09) | 0.19 | 0.29 | 0.77 |
Legend: OR: odds ratio by subgroups; significance level set for p-value under 0.05; MOF defined by low trauma fracture at hip, humerus, forearm and vertebral; Vertebral fractures: radiological grades 2-3 or 2 grade 1 fractures; non vertebral fracture: hip, humerus and forearm fractures; HGS: Handgrip Strength; ALM: Appendicular Lean Mass. EWGSOP: European Working Group on Sarcopenia in Older People; SDOC: Sarcopenia Definitions and Outcomes Consortium.
5
